# Surface roughness and microhardness of enamel white spot lesions treated with different treatment methods

**DOI:** 10.1016/j.heliyon.2023.e18283

**Published:** 2023-07-18

**Authors:** Mina MG. Chabuk, Abdulla MW. Al-Shamma

**Affiliations:** Department of Restorative and Aesthetic Dentistry, College of Dentistry, University of Baghdad, Baghdad, Iraq

**Keywords:** Bioactive glass, Microabrasion, Resin infiltration, White spot lesions, pH cycling

## Abstract

**Objective:**

To analyse the surface roughness and microhardness of artificial enamel white spot lesions before and after WSL formation, after treatment (Opalsutre™ microabrasion, Sylc® bioactive glass, and ICON® resin infiltration), and after pH cycling with the help of the profilometer surface roughness tester and the digital Vickers microhardness tester.

**Materials and methods:**

Seventy-five extracted molars were used to acquire one hundred specimens. 50 specimens were randomly assigned to five groups (n = 10) for the surface roughness study: 1) Sound group, 2) WSL group, 3) micro abrasion (MA; Opalustre, Ultradent, South Jordan, UT, USA), 4) bioactive glass 45S5 Sylc powder (Sylc; Denfotex Research Ltd, Inverkeithing, UK), and 5) ICON resin infiltration (ICON; DMG, Hamburg, Germany). An additional 25 specimens were used to obtain 50 enamel slabs for the surface microhardness study, which were also assigned to the same groups. All groups underwent a final stage of pH cycling. Surface roughness and surface microhardness measurements were performed at different stages for all groups.

**Results:**

Regarding surface roughness, ICON significantly reduced the surface roughness compared to Opalustre and Sylc, with no significant difference between Opalustre and Sylc. In terms of surface microhardness, ICON showed the highest improvement, followed by Sylc and then Opalustre. Both surface roughness and microhardness were significantly affected by demineralization, partially improved after treatment, and then regressed significantly after pH cycling.

**Conclusion:**

ICON resin infiltrant can be considered as a superior treatment option for improving surface roughness and microhardness, while Opalustre demonstrated relatively the poorest performance compared to the other treatment options. It is noteworthy that the pH cycling procedure had an adverse impact irrespective of the treatment option used.

## 1 Introduction

White spot lesion (WSL) is a clinical term used to describe an enamel surface and subsurface initial form of demineralization caused by plaque build-up in stagnation areas in individuals with poor oral hygiene. If this demineralization process is not halted, it ultimately progresses further into a cavity [[1], [2], [3]]. Aesthetics are not the only concern when it comes to WSLs, as they also affect the tooth's structural integrity since the demineralization process can increase the pore size of the enamel, harbouring more air and water volume in these pores [4]. In comparison to sound enamel, the white spot lesion's enamel is initially relatively hard, and it may or may not be rougher than the neighbouring sound enamel surfaces. Over time, the demineralization process jeopardizes the hardness of the WSL [5].

In the past, the treatment of WSLs included extensive removal of tooth structure through direct or indirect restoration. However, presently, more minimally invasive dental treatments (MID) are the preferred option [6]. Some of the MIDs used are: (a) microabrasion, which is based on the principle of micro-abrading the enamel surface with a slurry containing abrasive microparticles coupled with an acid, removing a thin layer of the discoloured enamel [7]; (b) bioactive glass (BAG), which is also a great candidate for enamel WSL remineralization, with a significant amount of hydroxyapatite (HAp) formation [[8], [9], [10], [11]]; and (c) resin infiltration, which relies on a low-viscosity resin that seeps into the porosities of non-cavitated lesions [12].

After meticulous research into the existing literature, to the best of our knowledge, this study is the first to determine the most effective treatment technique (Opalustre microabrasion, Sylc bioactive glass and ICON resin infiltration) in preserving surface roughness and microhardness at various stages (Sound, demineralization, treated WSL and after pH cycling). The null hypothesis assumes no significant difference in surface roughness and microhardness among the different stages and among the three treatment methods for enamel WSLs.

## 2 Materials and methods

This research project (Ref. number: 495) was approved by the research ethics committee of the College of Dentistry, University of Baghdad, Baghdad, Iraq, on 19-1-2022. The approval was granted based on the submission of informed consent forms from the patients, along with a thorough review of the application form, checklist, study protocol, and patient information sheet and consent, both in Arabic and in English.

### 2.1 Specimen preparation

A total of seventy-five human permanent mandibular first molars with sound buccal surfaces were collected for this study from various Iraqi dental clinics. Among these, fifty teeth were selected for the surface roughness study, while the remaining 25 teeth were used to create 50 enamel slabs for the surface microhardness study. Teeth that exhibited cracks, stains, fluorosis (inspected using a magnifying lens (Fukai, China) and a light curing unit), or developmental anomalies were excluded from the study [13,14].

The specimens were thoroughly washed and cleaned using tab water. To remove debris and soft tissue, they were polished with a non-fluoridated pumice-water suspension (Produits Dentaires SA, Vevey, Switzerland) using prophylactic brushes attached to a slow-speed handpiece. Next, the teeth were then immersed in a 0.1% Thymol solution (DR Thym™, Canada) for disinfection purposes and stored for 48 h. They were then kept in distilled water in a refrigerator at a temperature of 4 ± 0.1 °C until further use [15,16].

For the surface roughness study, the selected teeth were mounted in acrylic blocks measuring 25 mm in height and 20 mm in width. The mounting was done with the assistance of a surveyor to ensure that only the buccal surfaces of the teeth were exposed for analysis.

For the microhardness study, a total of fifty enamel slabs were obtained from the remaining 25 specimens using XP precision sectioning saw (Pelco, USA). Each enamel slab had dimensions of 2 mm in height, 4 mm in width, and 2 mm thickness. From each specimen, two slabs were taken from the buccal surface. Based on the findings of a pilot study, the flattest region on the buccal surface was identified for obtaining the two enamel slabs. This region was located 1 mm occlusal to the cervical ridge and extended 2 mm occlusally. The selected region was centrally positioned and spanned the surface mesiodistally. A full list of products names, compositions and sources have been provided in Table 1.

**Table 1:** Materials used in the current study.

Product	Composition	Approximate Wt.%/concentration	Source
Thymol solution	Thyme oil component		DR Thym™, InnuScience, Canada
Opalustre	Silicon carbide	>30-<50%	Ultradent, South Jordan, UT, USA
	Hydrochloric acid,	6.6%	
	Polyethylene Glycol	>1-<10%	
	Dimethicone	<1%	
	Trade Secret	>1-<10%	
Sylc	Silicon	21%	Denfotex Research Ltd, Inverkeithing, UK
	Calcium	18%	
	Sodium	18%	
	Phosphorus	3%	
	Oxygen	40%	
ICON	Hydrochloric acid	15–20%	DMG, Hamburg, Germany
	Ethanol; ethyl alcohol	55–99%	
	TEDMA	70–95%	
	Camphoro Quinone	<2.5%	
Methylcellulose	Methylcellulose	28.0–30.0%	Xi'a Geekee Biotech Co., Ltd, Shaanxi, China
	Hydroxypropyl	7.0–12.0%	
Lactic acid		0.1 M	LobaChemie Pvt. Ltd., Mumbai, India
Potassium hydroxide (KOH) solution		45%	HiMedia® laboratories, Pa, USA
Simulated oral fluid (SOF)	KCl	8.38 mM	
	MgCl2·6H2O	0.29 mM	
	CaCl2·2H2O	1.13 mM	
	K2HPO4	4.62 mM	
	KH2PO4	2.40 mM	
	Fluoride	0.22 ppm	
Remineralization solution (7 pH)	KCl	50 mmol/L	Pioneer Co., Sulaymaniyah, Iraq
	Ca	1.5 mmol/L	
	PO4	0.9 mmol/L	
	Trihydroxymethylaminomathane	20 mmol/L	
Demineralization solution (4.3 pH)	Ca	2 mmol/L	Pioneer Co., Sulaymaniyah, Iraq
	PO4	2 mmol/L	
	Acetate	0.075 mol/L	

### 2.2 Specimen grouping

For each study, the specimens were randomly assigned to the following groups:

Group I (sound): This group consisted of ten sound specimens.

Group II (WSL): Ten specimens underwent a demineralizing step to induce white spot lesions (WSL) without any further treatment.

Group III (Opal.): Ten specimens with existing WSL were treated using a microabrasion slurry (MA; Opalustre™, Ultradent, South Jordan, UT, USA).

Group IV (Sylc): Ten specimens with existing WSL were treated using Bioactive glass 45S5 Sylc therapeutic prophy powder (Sylc®; Denfotex Research Ltd, Inverkeithing, UK).

Group V (ICON): Ten specimens with existing WSL were treated using resin infiltration (ICON®; DMG, Hamburg, Germany).

**Fig. 1:**
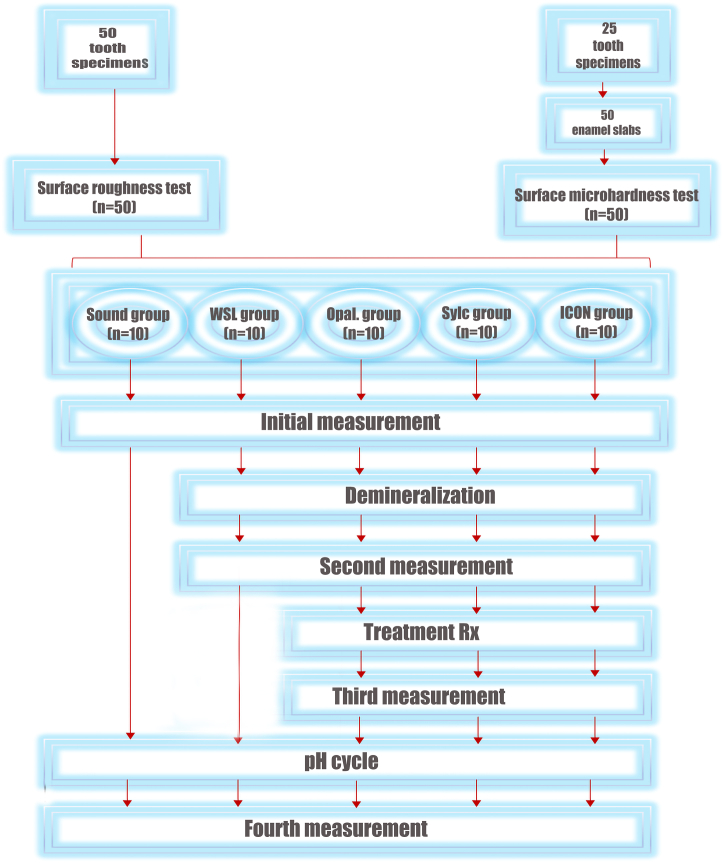
Illustration diagram of the study.

An illustration diagram of the study is presented in Fig. 1.

### 2.3 Demineralization procedure

The procedure involved the use of 8 g of methylcellulose gel (Xi'a Geekee Biotech Co., Ltd, Shaanxi, China). A demineralizing solution was prepared combining 100 ml of 0.1 M lactic acid (LobaChemie Pvt. Ltd., Mumbai, India), with a fixed pH of 4.6, adjusted using a 45% KOH solution (HiMedia® laboratories, Pa, USA) [17]. The mixture was then stored in an incubator at 37 °C for a period of 7 days. Throughout the incubation period, the pH was maintained at 4.5 using a digital pen-type pH meter (SD Fujian, China) [18].

### 2.4 Treatment approaches

#### 2.4.1 Microabrasion Opalustre™

Opalustre slurry is composed of 6.6% hydrochloric acid and silicon carbide microparticles ranging in size from 20 to 160 μm. The slurry is applied using Opalcups™ attached to a low-speed handpiece. The Opalcups™ are rubbed in a circular motion at a speed of 300 rpm for a duration of 30 s.

#### 2.4.2 Bioactive glass Sylc®

It is a dry powder composed of 100% bioactive glass, which is a calcium sodium phosphor-silicate material with particle size ranging from 25 to 120 μm. The application of the powder followed the manufacturer's recommendations, using a circular movement with an air pressure range of 40–46 psi. Each specimen was treated for 10 s, and the powder was left of the specimen for 60 min, followed by washing with running water, and a final 7 day soaking in simulated oral fluid (SOF), which was refreshed every 24 h, after these 7 days, the specimens were washed with de-ionized water and dried. This was done before conducting the measurements [19]. 8.38 mM KCl, 0.29 mM MgCl2·6H2O, 1.13 mM CaCl2·2H2O, 4.62 mM K2HPO4, 2.40 mM KH2PO4, and 0.22 ppm fluoride make up SOF's chemical composition. KOH was used to bring the pH value to 7.2, and the solution showed no precipitation throughout the experiment. The AquaCare air abrasion twin unit from VELOPEX International was used for the application of the Sylc powder.

#### 2.4.3 Resin infiltration ICON®

This material which was used in the study was applied following the instructions by the manufacturer. The process consisted of three steps:

First, Icon etch, which contains 15% hydrochloric acid, was applied to the tooth surface for a duration of 120 s. The tooth was then washed with water for 30 s and dried.

Second, Icon dry, a desiccating agent composed of 99% ethanol, was applied to the tooth surface for 30 s.

Lastly, Icon resin infiltrant, which is a methacrylate-based resin matrix, was massaged onto the surface of the lesion using the provided tip. The infiltrant was allowed to penetrate for 180 s and then light cured for 40 s.

### 2.5 pH cycling

The remineralization solution used in the study consisted of potassium chloride (KCl) (PiONEER Co., Sulaymaniyah, Iraq), calcium (Ca) (PiONEER Co., Sulaymaniyah, Iraq), and phosphate (PO4) (PiONEER Co., Sulaymaniyah, Iraq), at concentrations of 50 mmol/L KCl, 1.5 mmol/L Ca, and 0.9 mmol/L PO4, in a trihydroxymethylaminomathane (PiONEER Co., Sulaymaniyah, Iraq), buffer with a pH of 7. On the other hand, the demineralization stage involved an acid buffer containing 2 mmol/L Ca, 2 mmol/L PO4, and 0.075 mol/L acetate (PiONEER Co., Sulaymaniyah, Iraq), with a pH of 4.3.

Each tooth sample was soaked in 15 ml of the demineralizing solution for a period of 6 h, followed by rinsing with deionized distilled water. Subsequently, the tooth samples were soaked in 15 ml of the remineralizing solution for 18 h. This cycle was repeated over a period of 14 days, comprising 10 daily cycles [17].

### 2.6 Surface roughness evaluation

The surface roughness of the specimens was measured using a calibrated mechanical 2D profilometer surface roughness tester (leeb 432 A, leeb instrument Co. Ltd., Chongqing, China). The measurement of Ra (average roughness) was conducted using a diamond stylus with a diameter of 5 μm, mounted in a detector. The stylus moved perpendicularly onto the surface, covering a length of 1.25 mm, with a 0.25 mm cut-off length, applied a force of less than 0.004 N, and an average of three reading were taken for each specimen [20]. Surface roughness measurements were taken for each specimen at four different stages throughout the study.

### 2.7 Surface microhardness evaluation

**Fig. 2:**
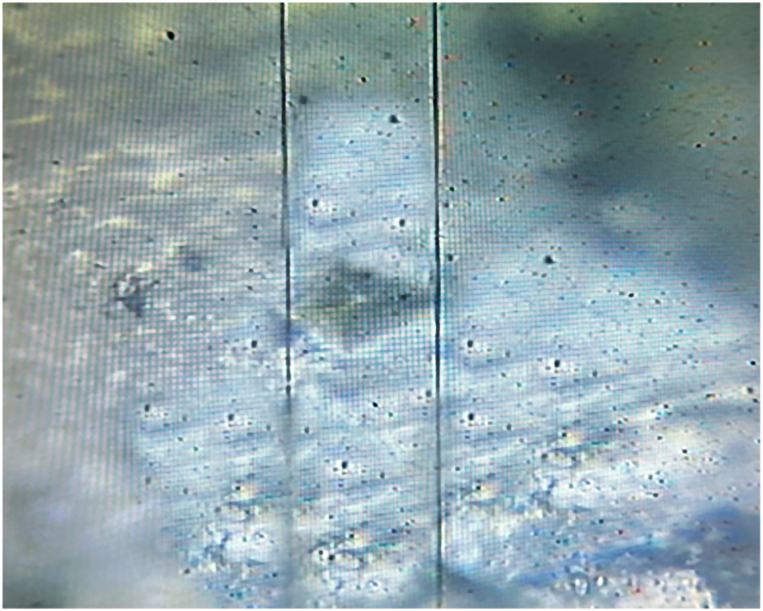
Screen image of the Vickers microhardness indentation.

The microhardness of the enamel slabs was measured using a Digital Vickers microhardness tester machine (Model: HVS-1000, laryee Technology Co., Ltd., Beijing, China). A load of 100 g was applied for a duration of 15 s. The resulting indentations were scanned with a 400× microscopic lens, and an average of three readings was calculated for each enamel slab, these readings were taken at locations 100 μm apart from each other. To measure microhardness, each enamel slab was divided into 4 quadrants representing each stage. In Fig. 2, the screen image of the Vickers microhardness indentation is shown.

### 2.8 Statistical analysis

Data description, analysis, and presentation were conducted using Statistical Package for Social Science (SPSS) version 22. The statistical analyses included the Shapiro-Wilk test for assessing the normality distribution of the data. One-way and repeated measure analysis of variance (ANOVA) were used for comparisons among groups. Tukey's honestly significant difference (HSD) test was employed as a post hoc test to compare subgroups. Additionally, a paired *t*-test was used to analyse differences between the two sound subgroups. The significance level was set at p < 0.05.

## 3 Results

### 3.1 Surface roughness

**Fig. 3:**
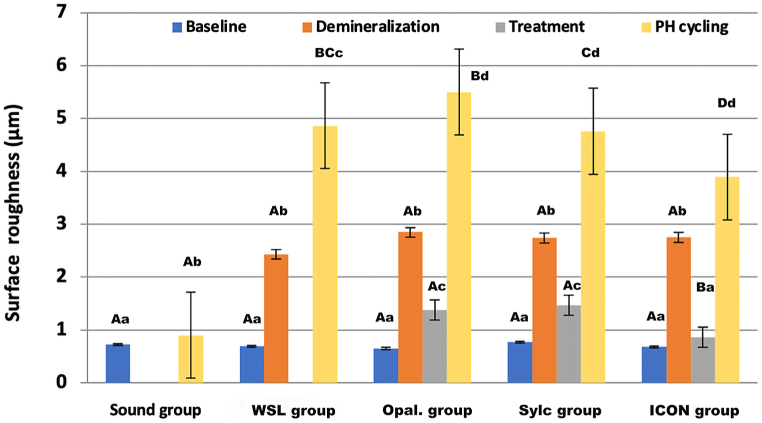
Bar chart demonstrating the differences in surface roughness among groups and stages. The uppercase letters indicate differences among groups at each stage, while lowercase letters indicate stage differences within each group.

The means, standard deviations, and inferential statistical data for the surface roughness part of this study are presented in Table 2 and Fig. 3. From the data depicted in Tables 2 and it can be observed that there were no statistically significant differences among groups at base line. After the demineralization stage, there was a statistically significant increase in surface roughness for all four demineralized groups, with no significant differences among them. However, after treatment, the surface roughness significantly decreased for the three treatment groups. Among the treatment groups, the ICON group exhibited the lowest surface roughness value (0.866 ± 0.203) μm, which was statistically significant compared to the other treatment groups, but not significantly different from the base line of the ICON group. The Opal. group followed with a surface roughness value of (1.379 ± 0.331) μm, which was not statistically significantly different from the Sylc group, which exhibited the highest surface roughness value of (1.473 ± 0.350) μm.

**Table 2:** Descriptive and statistical analysis of surface roughness average roughness Ra (μm) among groups and stages.

	Sound group (n = 10)	WSL Group (n = 10)	Opal. group (n = 10)	Sylc group (n = 10)	ICON group (n = 10)
Baseline	0.725 ± 0.096^Aa^	0.691 ± 0.163^Aa^	0.651 ± 0.152^Aa^	0.769 ± 0.182^Aa^	0.681 ± 0.155^Aa^
Demineralization		2.436 ± 0.551^Ab^	2.850 ± 0.499^Ab^	2.740 ± 0.564^Ab^	2.751 ± 0.63a^Ab^
Treatment			1.379 ± 0.331^Ac^	1.473 ± 0.350^Ac^	0.866 ± 0.203^Ba^
pH cycling	0.901 ± 0.220^Ab^	4.863 ± 0.998^BCc^	5.502 ± 0.555^Bd^	4.760 ± 0.600^Cd^	3.894 ± 0.392^Dd^

Statistically significant differences in data are represented with differences in initials. The uppercase letters demonstrating row differences, while lowercase letters demonstrating column differences (p > 0.05).

WSL: White spot lesion.

After undergoing pH cycling, all groups demonstrated a further significant increase in surface roughness. The Sound group exhibited the lowest value for surface roughness (0.901 ± 0.220) μm, which was statistically significantly lower compared to the other groups. The WSL group was not statistically significant when compared to the Opal. and Sylc groups but was statistically significant when compared to the ICON group. Among the treatment groups, they were all statistically significantly different from one another. The ICON group had the lowest value for surface roughness (3.894 ± 0.392) μm, followed by the Sylc group (4.760 ± 0.600) μm, and the highest value for the Opal. group (5.502 ± 0.555) μm.

### 3.2 Microhardness

**Fig. 4:**
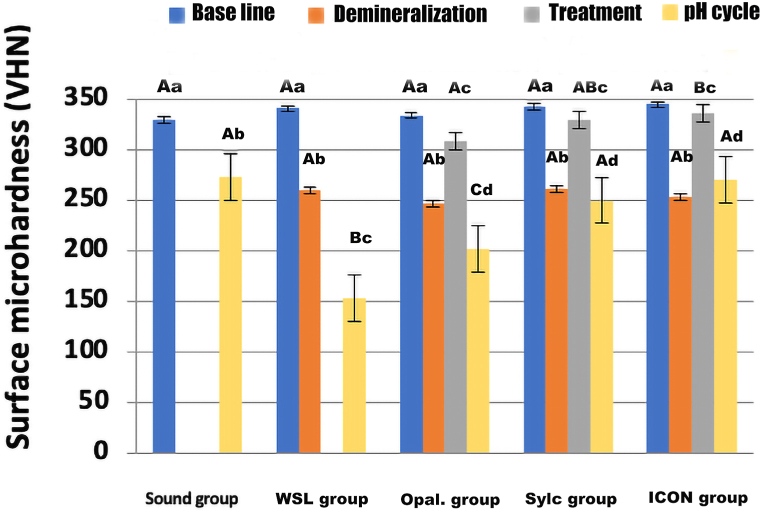
Bar chart demonstrating the differences in microhardness among groups and stages. The uppercase letters demonstrate differences among groups at each stage, while lowercase letters demonstrate stage differences within each group.

The statistical data pertaining to surface microhardness is presented in Table 3 and Fig. 4. It can be observed that there were no statistically significant differences among groups at base line. The microhardness significantly decreased in all four groups that underwent the demineralization stage, with no statistically significant differences among them. However, after the treatment stage, the microhardness significantly increased again for the three treatment groups. Among the treatment groups, the ICON group exhibited the highest value for microhardness (336.377 ± 23.958) VHN, which was statistically insignificant when compared to the Sylc group (329.757 ± 17.332) VHN, but statistically significant when compared to the Opal. group (307.660 ± 24.884) VHN. There were no significant differences between the Opal. and Sylc groups.

**Table 3:** Descriptive and statistical analysis of surface microhardness (VHN) among groups and stages.

	Sound group (n = 10)	WSL group (n = 10)	Opal. group (n = 10)	Sylc group (n = 10)	ICON group (n = 10)
Baseline	329.020 ± 13.225^Aa^	340.680 ± 15.894^Aa^	333.843 ± 21.010^Aa^	342.597 ± 19.598^Aa^	344.577 ± 24.274^Aa^
Demineralization		259.720 ± 19.070^Ab^	245.903 ± 26.159^Ab^	260.307 ± 24.360^Ab^	252.581 ± 27.650^Ab^
Treatment			307.660 ± 24.884^Ac^	329.757 ± 17.332^ABc^	336.377 ± 23.958^Bc^
pH cycling	273.207 ± 14.646^Ab^	153.257 ± 21.634^Bc^	201.380 ± 21.180^Cd^	249.623 ± 19.366^Ad^	270.237 ± 24.750^Ad^

Different initials depict statistically significant differences in surface microhardness among groups and stages. Row differences are depicted in Uppercase letters, while differences in columns are represented in lowercase letters (p > 0.05).

WSL: White spot lesion.

Following the pH cycling procedure, the surface microhardness once again significantly decreased for all groups. The WSL group enamel microhardness (153.257 ± 21.634) VHN was significantly lower compared to all other groups. The Opal. group microhardness also significantly decreased compared to the sound and other treatment groups. The other treatment groups (Sylc and ICON) and the sound group produced the highest microhardness results, with no significant difference between each pair of them.

## 4 Discussion

Studies have shown that sound dental enamel typically has a surface roughness ranging between 0.59 μm and 0.66 μm [21]. However, after the initiation of WSL, it was observed that the surface roughness significantly increased in all groups, except for the sound group. This observation is consistent with previous studies, such as the one conducted by Kielbassa et al. , which revealed the presence of cavitation in the pseudo-intact surface layer covering the WSL when examined under an electron microscope [22]. The increase in surface roughness can be attributed to the dissolution of hydroxyapatite crystals during the demineralization process, which leads to the formation of microporosities in the superficial layer. This mineral loss caused by the slow acidic action contributes to the erosion in the superficial layer [23,24]. These findings align with the research conducted by Hussein and Al-Shamma [25] and I'udzuri [26].

All treatment options investigated in this study demonstrated the ability to reduce the surface roughness of the artificial WSL. However, it should be noted that none of the treatment groups were able to completely restore the surface roughness to the baseline level, except for the ICON treated group.

In the Opal. group, the product contains two main components that exert distinct actions. The hydrochloric acid component contributes to an erosive action, while the silicon carbide particles contribute to an abrasive action. Together, these components work synergistically to remove the rough and porous enamel surface [7,27]. These findings are consistent with the study conducted by El Sayed and Fahmy [28]. Considering these results, it can be concluded that Opalustre treatment was effective in reducing the surface roughness of the artificial WSL, although it did not fully restore the enamel to its baseline state. The combined erosive and abrasive actions of Opalustre appear to play a role in the smoothing and levelling of the enamel surface, as supported by previous studies.

In the Sylc treated group, there is limited information available regarding the specific analysis of surface roughness using Sylc in treating the artificial WSL. However, the mechanism of action of Sylc can provide some insights into its potential effects on surface roughness. Sylc is composed of calcium sodium phosphocilicate bioactive glass particles, which have the ability to undergo a surface reaction when in contact with an aqueous solution. This reaction results in the formation of Hydroxy Carbonate apatite (HCA), a calcium and phosphate rich layer that precipitates on the enamel surface [17,29]. The Bioactive glass particles physically and chemically adhere to the enamel porosities and block their passageways, leading to the filling of the empty microporosities in the WSL surface layer [[30], [31], [32]]. In this study, it was found that the surface roughness was significantly improved after using Sylc. This improvement could be attributed to the formation of HCA crystals, which effectively fill the microporosities present in the WSL surface layer. By filling these microporosities, the Sylc treatment likely contributes to the increased smoothness of the enamel surface, resulting in an improvement in surface roughness.

In ICON treated group, the surface roughness of enamel was significantly reduced to a level comparable to sound enamel, which is consistent with the findings of Aswani et al. [33]. This can be explained by how ICON typically works. During the ICON treatment, the enamel surface is first etched using a hydrochloric acid gel. This etching process efficiently erodes the surface and opens up the porosities present in the WSL. The low viscosity resin material used in the treatment then flows into these opened porosities and undergoes polymerization, causing a complete obliteration of the microporosities. This mechanism of action leads to the development of a smooth enamel surface. The resin infiltrate effectively fills the microporosities, resulting in a surface that closely resembles sound enamel. It is worth noting that there are some conflicting findings in the literature regarding the achievement of baseline surface roughness with the ICON treatment. Studies by Yazkan and Ermis, and Almulhim et al. reported an improvement in surface roughness but did not reach the level of sound enamel. This discrepancy could be attributed to microscopic amounts of resin infiltrate accumulating in certain regions on the enamel surface or in some irregularly etched regions. These localized irregularities may contribute to a slightly higher surface roughness measurements compared to sound enamel. Overall, ICON treatment has shown promising results in reducing surface roughness and achieving a surface that closely resembles sound enamel. However, further studies are needed to explore the potential variations in surface roughness outcomes and to optimize the application technique for more consistent results [27,34].

In all groups, the pH cycling challenge resulted in a significant increase in surface roughness [35,36]. This is thought to be the result of mineral loss and alterations in the porosity of the enamel surface that occur during the pH cycling process [[37], [38], [39]]. During pH cycling, the enamel surface experiences cycles of demineralization and remineralization, mimicking the conditions in the oral environment. This process can lead to further mineral loss from the enamel and changes in the porosity of the surface, which can contribute to an increase in surface roughness. The observed inadequate surface roughness in this stage may be explained by the inability of the enamel prisms to regain a regular repositioning of ions between them. This phenomenon was observed in a study by Willmot [40], which examined the topographic features of the enamel surface and demonstrated the presence of a regular periodicity of Retzius lines. Disruptions in this regular periodicity may result in irregularities and variations in surface roughness. The same explanations can be applied to the Opal. group, where the pH cycling challenge also led to an increase in surface roughness.

In the Sylc treated group, to the author's knowledge, no studies have been conducted using sylc for treating white spot lesions followed by a pH cycling procedure. It is important to note that during an ionic exchange, the properties of the hydroxyapatite (HAp) crystals can undergo changes. For instance, the hydroxy group may be replaced with a carbonate group, increasing the solubility of the crystals and leading to the formation of microporosities. Consequently, any substitution within the crystal lattice directly affects the behaviour of the apatite crystals, particularly in low pH environments [15,41]. Additionally, the HAp crystals formed after treatment with Sylc are amorphous, as opposed to the highly crystalline rod-shaped HAp crystals. Amorphous HAp crystals are more susceptible to acid dissolution compared to their highly crystalline counterparts [42].

In the ICON treatment group, one of the main components of ICON is triethylene glycol dimethacrylate (TEGDMA), has a high hydrophilicity, which makes it prone to water sorption, hydrolysis, and elution in water-based materials [43]. ICON is known to have a low conversion degree of approximately 50% [42] and its lack of filler content further contributes to these conditions [44]. The elution of monomers is a phenomenon commonly found in composites, adhesives, and other resin-based materials, is seen to be extensively less than that found in ICON treated lesions [35,36,45,46]. It is worth noting that different results were obtained by Wierichs [47], where ICON was found to resist the pH cycle and prevent further lesion progression. These differences in findings could potentially be attributed to variations in the pH cycling conditions employed in the studies.

The findings of our study reveal a noteworthy increase in surface roughness of artificial enamel WSLs following pH cycling. This raises concerns about surface quality and motivates us to investigate potential strategies for improvement. To address this issue, adjustments to the pH cycling conditions, such as modifying the duration, frequency, or the specific solutions used, may help minimize the negative impact on the surface roughness. Additionally, exploring alternative treatment protocols, for example; remineralization agents or novel approaches aimed at enhancing the surface smoothness, could provide potential solution.

In the microhardness study, the initiation of WSLs led to a significant decrease in microhardness in all the groups that underwent demineralization [48].

In the Opal. Treated group, the surface microhardness was statistically significantly improved, which can be attributed to the compacting effect of the acidic component on the enamel prismatic structure, resulting in a prism free area [7,49]. Another possible explanation is within the mechanism of action of opalustre, which involves removing the weakened and porous superficial enamel layer, exposing a harder sound enamel layer. However, the surface microhardness of this group did not reach the baseline, which contradicts the findings of Yazkan and Ermis [27] where the surface microhardness of the microabrasion group matched the baseline levels.

In the Sylc treated group, the surface microhardness was measured after a 7-day soak in SOF, which revealed a significantly improved surface microhardness compared to the demineralization stage. This is consistent with the findings of Taher and Haridy, and Ahmed [50,51]. The remineralization primarily occurs on the surface of the WSL, where the superficial pores and pathways are effectively filled with the precipitated material, resulting in the formation of a chemically and physically attached hydroxyapatite (HCA) layer [52]. Calcium and phosphate nanoparticles formed within the demineralized subsurface contributes to the arresting of demineralization and the enhancement of remineralization, thereby improving enamel microhardness [50]. A study by Dong et al. where three different types of bioactive glasses one of which is the 45S5 bioactive glass were examined, demonstrated the filling of enamel porosities with bioactive glass particles and subsequent deposition of ovoid HAp crystals after a 7 day soak in SOF, a thick mineralized layer had formed with an average thickness of 4 μm for the 45S5 bioactive glass [19]. The hardness of Sylc glass material (4.5–5.75 GPa), surpasses that of enamel (3.5 GPa) [40], further contributing to the observed improvement in microhardness. In conclusion, the enhanced microhardness after treatment with Sylc can be attributed to the incorporation of HAp crystals into enamel porosities, rather than solely the mineralized layer formed. It is important to note that the microhardness test assesses the entire enamel structure, not just the superficial part affected by the WSL, emphasizing the overall improvement achieved through this treatment method.

As for the ICON treated group, the surface microhardness significantly increased after treatment, for the reasons explained previously. However, the microhardness did not reach the level of the baseline, which is consistent with the findings of Neres et al. [35]. This could be related to the fact that certain areas in the demineralized enamel remaining non-infiltrated during polymerization, possibly due to material shrinking [53]. The polymer chains of the (TEGDMA) monomer, which is the main component of the organic matrix of the ICON resin infiltrate, may not always form optimally [[54], [55], [56], [57], [58]]. Additionally, the mechanical properties of this monomer are less favourable compared to other monomers, as it lacks aromatic rings and strong intermolecular secondary bonds [40]. These factors may contribute to the surface microhardness of the ICON treated group not reaching that of sound enamel.

After pH cycling, all groups were once again unable to maintain their hardness values, with a significant drop in surface microhardness, considering the previously mentioned reasons [35,36,45]. This finding underscores the complexity of the changes occurring and necessitates further investigation. To address the increase in surface microhardness, it is crucial to analyse the underlying factors contributing to this outcome. It may be beneficial to assess the impact of the pH cycle on the mineral composition and crystalline structure of enamel. by gaining a deeper understanding of these changes, we can identify targeted interventions to manage the surface microhardness increase effectively. Additionally, evaluating the effectiveness of different cleaning or polishing techniques and exploring the application of protective coatings or surface modifiers can help maintain the desired balance between surface hardness and overall surface quality.

Further research and experimentation are needed to validate the effectiveness of these approaches and enhance our understanding of the underlying mechanisms involved.

## 5 Study limitations

There are a few limitations to be aware of in the present study.

1. This is an in-vitro study model, and with these models, it is difficult, if not impossible, to perfectly mirror the natural oral environmental conditions, such as the salivary flushing actions and clearance.
2. The use of artificial lesions may not fully capture the complexity and characteristics of natural enamel lesions.
3. A limited focus on specific treatments, this study primarily focuses on Opalustre, Sylc and ICON, other potential treatment options may be explored.
4. The study's sample size and duration may impact the statistical power to detect smaller or more nuanced differences and might not fully capture long-term effects or changes that occur over extended time periods.

## 6 Conclusion

The ICON resin infiltrant appeared to provide the most favourable outcome in terms of surface roughness and microhardness compared to the other treatment groups. However, it is worth noting that the surface roughness and microhardness of all the groups deteriorated after pH cycling.

## Funding information

This research project was self-funded.

## Ethical approval

This research project was approved by the research ethics committee of the College of Dentistry, University of Baghdad, Baghdad, Iraq. Ref. number: 495. Date: 19-1-2022.

## Author contributions

Conceptualization: Mina M. Chabuk, Abdulla M.W. Al-Shamma; Methodology: Mina M. Chabuk, Abdulla M.W. Al-Shamma; Software: Mina M. Chabuk; Validation: Mina M. Chabuk, Abdulla M.W. Al-Shamma; Formal Analysis: Mina M. Chabuk, Abdulla M.W. Al-Shamma; Investigation: Mina M. Chabuk, Abdulla M.W. Al-Shamma; Resources: Abdulla M.W. Al-Shamma, Ban Al-Ghadhanfari, Yassir Al-Khannaq; Data Curation: Mina M. Chabuk, Abdulla M.W. Al-Shamma; Writing – original draft preparation: Mina M. Chabuk; Writing – review and editing: Mina M. Chabuk, Abdulla M.W. Al-Shamma; Visualization: Mina M. Chabuk, Abdulla M.W. Al-Shamma; Supervision: Abdulla M.W. Al-Shamma; Funding acquision: Mina M. Chabuk.

## Declaration of competing interest

The authors declare that they have no known competing financial interests or personal relationships that could have appeared to influence the work reported in this paper.
